# De novo paternal origin duplication of chromosome 11p15.5: report of two Chinese cases with Beckwith-Wiedemann syndrome

**DOI:** 10.1186/s13039-017-0347-z

**Published:** 2017-12-19

**Authors:** Qin Wang, Qian Geng, Qinghua Zhou, Fuwei Luo, Peining Li, Jiansheng Xie

**Affiliations:** 1 Shenzhen Maternity and Child Healthcare Hospital, 3012 Fuqiang Road, Shenzhen, Guangdong 518028 China; 2 First Affiliated Hospital, Biomedical Translational Research Institute, Jinan University, Guangzhou, Guangdong China; 3 Department of Genetics, Yale School of Medicine, New Haven, CT USA

**Keywords:** Beckwith–Wiedemann syndrome (BWS), Chromosome 11p15.5, Imprinting centers (IC), Paternal duplication, Chromosomal microarray analysis(CMA), Methylation-specific multiplex ligation-dependent probe amplification (MS-MLPA)

## Abstract

**Background:**

The molecular etiology of Beckwith-Wiedemann syndrome (BWS) is complex and heterogeneous. Several subtypes of epigenetic-genetic alterations including aberrant methylation patterns, segmental uniparental disomy, single gene mutations, and copy number changes have been described. An integrated molecular approach to analyze the epigenetic-genetic alterations is required for accurate diagnosis of BWS.

**Case presentation:**

We reported two Chinese cases with BWS detected by genome-wide copy number analysis and locus-specific methylation profiling. Prenatal analysis on cord blood of patient 1 showed a de novo paternal origin duplication spanning 896Kb at 11p15.5. Patient 2 was referred at 2-month old and the genetic analysis showed a de novo 228.8Kb deletion at 11p15.5 telomeric end and a de novo duplication of 2.5 Mb at 11p15.5–15.4. Both the duplications are of paternal origin with gain of methylation at the imprinting center 1 and thus belong to the subgroup of a low tumor risk.

**Conclusion:**

Results from these two cases and other reported cases from literature indicated that paternally derived duplications at 11p15.5 region cause BWS. Combined chromosome microarray analysis and methylation profiling provided reliable diagnosis for this subtype of BWS. Characterization of genetic defects in BWS patients could lead to better understanding the genetic mechanisms of this clinically and genetically heterogeneous disorder.

## Background

Human chromosome 11p15.5 contains a one megabase (Mb) cluster of evolutionary conserved imprinted genes [1]. The expression of these imprinted genes is regulated by two different imprinting centers (IC) 1 and 2. Epigenetic and genetic alterations affecting chromosome 11p15.5 cause Beckwith-Wiedemann syndrome (BWS,OMIM#130650), which is a congenital overgrowth disorder with an incidence of about one in 13,000. BWS has a complex phenotype typically including prenatal and postnatal overgrowth, macroglossia, exomphalos, umbilical hernia, hemihypertrophy, and hypoglycaemia in the neonatal period. Affected patients also have increased risk for developing tumors in childhood, including Wilm’s tumor, adrenal cortical carcinomas, and hepatoblastoma [2]. However, the frequency of tumor occurrence in BWS patients have varied from studies. Molecular genetic diagnosis would facilitate the subtyping of BWS and identifies individuals who require the surveillance due to increased risk of pediatric tumors.

The molecular etiology of BWS is complex and several subtypes have been described. Up to 80% of sporadic cases are due to epigenetic-genetic alterations and about 15–20% BWS patients do not have a known genetic defect. About 85% of BWS cases occur sporadically and 15% have familial transmission. Among BWS patients with a defined genetic defect, 50% are caused by hypomethylation of IC2 leading to loss of methylation at the *KCNQ1OT1:TSS DMR* (differentially methylated region), 5–10% by hypermethylation of *H19/IGF2:IG DMR* resulting in bi-allelic expression of the *IGF2* (insulin like growth factor 2) gene and absence of *H19* gene expression, 20% by aberrant methylation patterns on both imprinted gene clusters due to segmental paternal uniparental disomy (UPD) covering 11p15, 8% by germline *CDKN1C* mutations, and about 1–2% by a paternal duplication or a balanced rearrangement of chromosome 11p15 [2–5]. Most of the duplication cases result from unbalanced segregation of a paternal translocation or inversion. However, paternal derived de novo duplications involving 11p15.5 have also been described [2, 3]. The identified duplication is predicted to affect the dosage and expression of paternally expressed genes including overexpression of *IGF2* gene, as well as nonimprinted genes. An integrated molecular approach is required to provide accurate diagnosis of BWS. Proper subtyping of epigenetic-genetic alterations in BWS patients could lead to better understanding of disease course, effective treatment of symptoms, and preventive management of tumor risk.

## Case presentation

Two patients were referred for genetic evaluation at the prenatal diagnosis and genetic counseling clinic in Shenzhen Maternal and Child Healthcare Hospital. This study was approved by the hospital’s Institutional Review Board and written informed consents for publication of their clinical details and/or clinical images were obtained from the patient or parents.

**Fig. 1:**
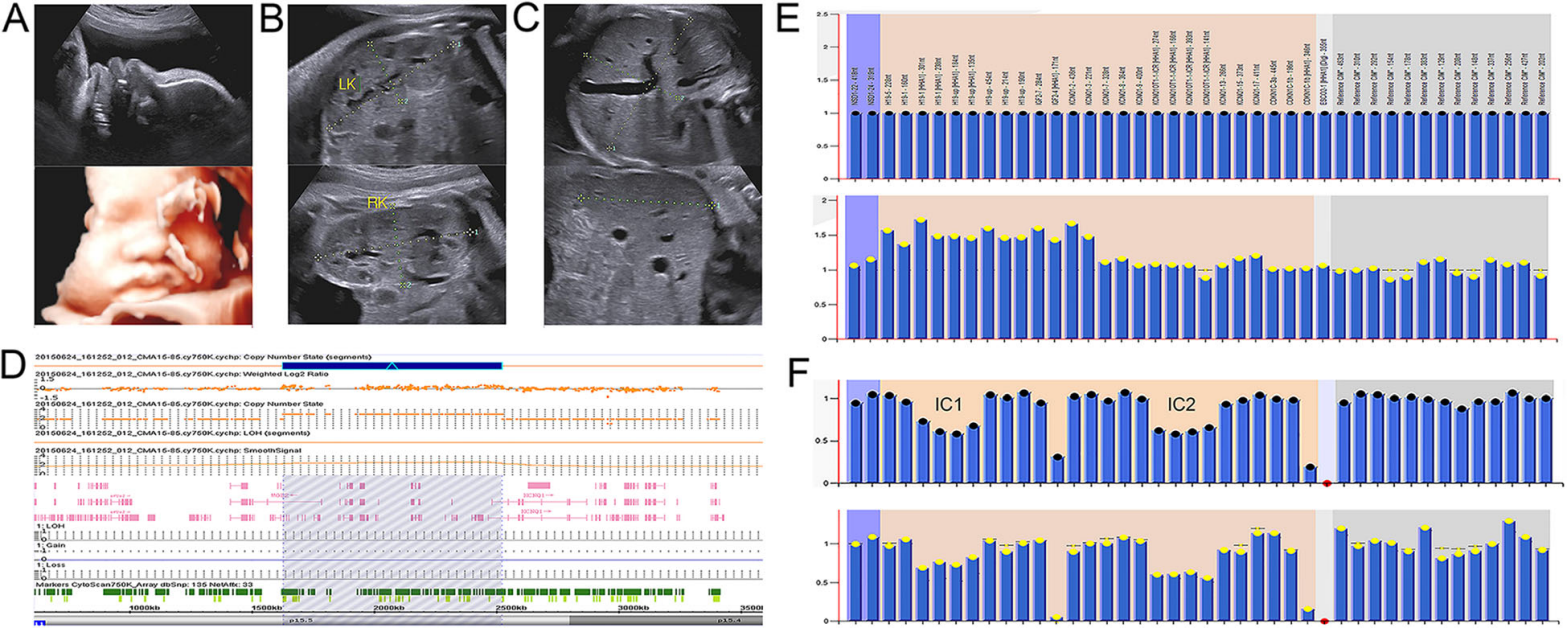
Prenatal ultrasound images at 34 weeks of gestation, CMA and MS-MLPA results in patient 1. **a** 2D and 3D prenatal ultrasound examinations at 34 weeks demonstrated macroglossia. **b** Nephromegaly at 34 weeks prenatal ultrasound image with left kidney length of 7.89 × 3.39 × 3.67 cm, right kidney length of 6.86 × 3.59 × 3.55 cm. **c** Hepatomegaly at 34 weeks prenatal ultrasound image with length of 5.46 × 8.18 × 5.28 cm. **d** The CMA chromosome view (up) and gene view (bottom) reveal the breakpoint location and an 896Kb duplication at 11p15.5 (arr[GRCh37]11p15.5(1,632,167–2,527,910)×3). **e** MS-MLPA shows a peak height ratio value of 1.5 (three copies) at 11p15 (bottom) in comparison with a ratio value of 1 (two copies) from a normal control (upper). **f** MS-MLPA indicates methylation index of 0.76 at IC1 and methylation index of 0.61 at IC2 (bottom) in comparison with normal control methylation index of 0.65 at IC1 and 0.62 at IC2 (upper)

Patient 1 was a 27-year-old G_1_P_0_ woman with no significant past medical, surgical or family history. Fetal ultrasound screening in the 24 weeks of gestation was unremarkable except for mild enlargement of renal. By week 30, ultrasonography examination identified a protruding tongue and enlargement kidney and liver. The follow-up ultrasound study by week 34 confirmed macroglossia, nephromegaly and hepatomegaly in the fetus (Fig. 1a–c). Polyhydramnios (AFI = 22.26 cm, 75th -95th percentile), increased abdominal circumference (AC = 34.34 cm, 75th -95th percentile) and an increased estimated fetal weight (EFW = 3228 g, >95th percentile) were also noted.

**Fig. 2:**
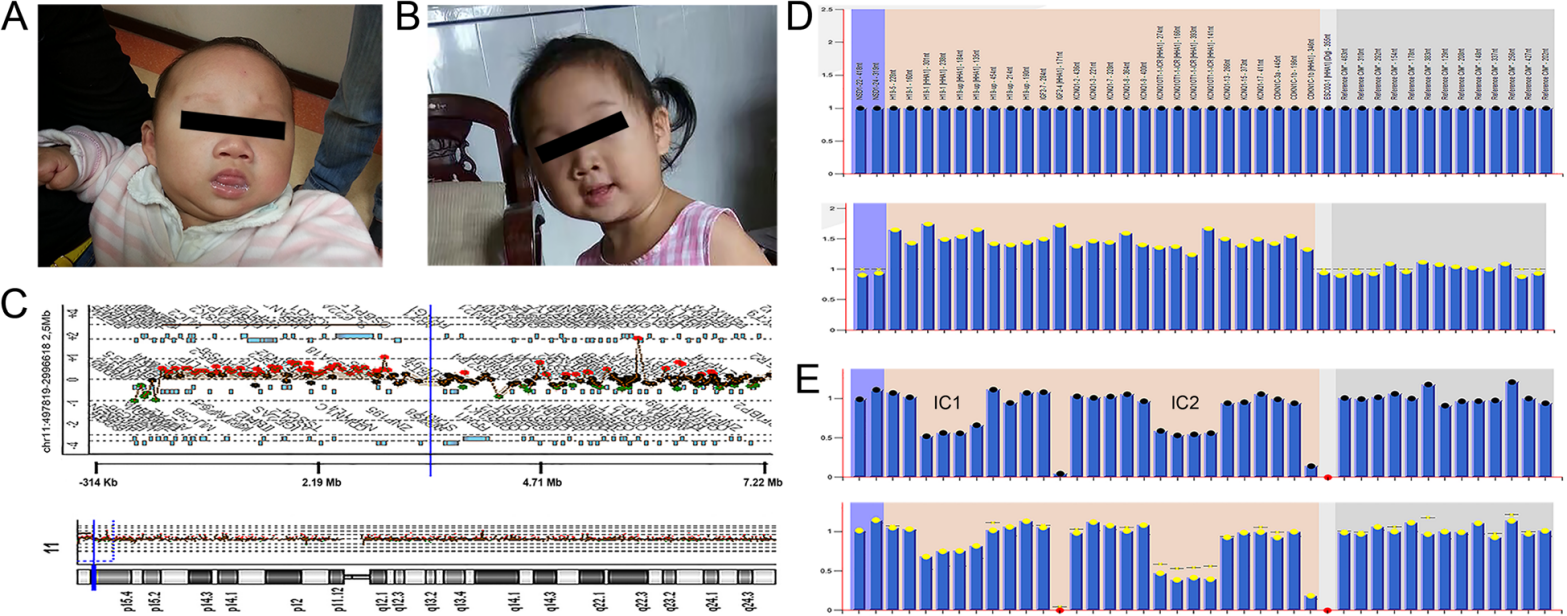
Appearance at 2 months and 32 months of age, aCGH and MS-MLPA results in patient 2. **a** Macroglossia was noted at 2 months of age. **b** Appearance at 32 months showed reduced macroglossia with age. **c** The aCGH result shows a 228.8Kb deletion at 11p15.5 and a 2.5 Mb duplication at 11p15.5–15.4 (arr[GRCh36] 11p15.5(208,165–436,954)×1,11p15.5p15.4(497,819–2,996,618)×3). **d** MS-MLPA shows a peak height ratio value of 1.5 (three copies) at 11p15 (bottom) in comparison with a ratio value of 1 (two copies) from a normal control (upper). **e** MS-MLPA indicates IC1 methylation index of 0.75 and IC2 methylation index of 0.42 (bottom) in comparison of normal control methylation index of 0.58 at IC1 and 0.56 at IC2 (upper)

Patient 2 was a 2-month-old baby girl with weight 4.35 kg (15th percentile) and length 53.5 cm (under 15th percentile). She came from a rural country of Guangdong province. Her mother did not have prenatal tests during the pregnancy, no miscarriages history and the family had no medical history of BWS. After birth, she showed macroglossia, earlobe crease, hypermyotonia and had feeding and airway issues. The macroglossia was evident at 2 months of age (Fig. 2a). Patient 2 was able to eat and speak normally and has reduced macroglossia at 32 months old (Fig. 2b). Physical examination at local hospital by physician showed normal development (weight 13.2 kg, 50th percentile), height 91 cm, < 50th percentile) and no abnormal ultrasound examination results were found.

In patient 1, chromosome analysis showed a normal male karyotype (46,XY). Chromosome microarray analysis (CMA) was performed for patient 1 using the Affymetrix Cytoscan 750 K array (Affymetrix, Santa Clara, CA, USA) by following the manufacturer’s instructions. The CMA result revealed an 896 k-base (Kb) duplication at 11p15.5 (arr[GRCh37]11p15.5(1,632,167–2,527,910)×3) including genes from *CTSD* to *TRPM5* (Fig. 1d). The MS-MLPA (Amsterdam, The Netherlands; SALSA MLPA kit ME030-B2) result showed copy number changes with an increased mean peak height ratio of 1.5. This was interpreted as a trisomic pattern for the 11p15 IC1 region and exclude the duplication of the IC2 region (Fig. 1e). The methylation status was gain of methylation at IC1 with a methylation index of 0.76 and normal methylation status on IC2 with a methylation index of 0.61 in comparison with normal control methylation index of 0.65 at IC1 and methylation index of 0.62 at IC2 (Fig. 1f). Six normal cord blood control samples were analyzed for patient 1. Normal methylation ranges at IC1 and IC2 were calculated from the control samples. The IC1 and IC2 methylation indices were referred to the established ranges [6]. After comprehensive counseling of the genetic test results and associated disease phenotypes, the couple made an informed decision to terminate the pregnancy. Follow-up chromosome analysis, CMA and MS-MLPA performed for the couple found normal results.

In patient 2, chromosome analysis showed a normal female karyotype (46,XX). Array comparative genomic hybridization analysis was performed for patient 2 using the SurePrint G3 Human CGH 8x60K Microarray Kit (Agilent Technologies, Santa Clara, CA, USA) by following a previous described procedure [7]. The aCGH result showed a 228.8Kb deletion at 11p15.5 including genes from *SIRT3* to *ANO9* and a 2.5 Mb duplication at 11p15.5–15.4 including genes from *HRAS* to *CARS* (arr[GRCh36] 11p15.5(208,165–436,954)×1,11p15.5p15.4(497,819–2,996,618)×3) (Fig. 2c). The MS-MLPA (Amsterdam, The Netherlands; SALSA MLPA kit ME030-B2) result showed copy number changes with an increased mean peak height ratio of 1.5. This was interpreted as a trisomic pattern for this chromosomal region (Fig. 2d). The methylation status was gain of methylation at IC1 with methylation index of 0.75 and normal methylation status at IC2 with methylation index of 0.42 in comparison with normal control methylation index of 0.58 at IC1 and methylation index of 0.56 at IC2 (Fig. 2e). Ten normal peripheral blood control samples were analyzed for patient 2. Follow-up parental chromosome analysis, CMA and MS-MLPA found normal results.

## Discussion

In this study we report two Chinese BWS cases with de novo paternally derived duplication at 11p15.5. For patient 1, the findings of macroglossia, nephromegaly and hepatomegaly by ultrasound examinations and the results from CMA and MS-MLPA analysis fulfilled the diagnostic criteria for BWS with low tumor risk. Currently, there have been more than 150 BWS cases diagnosed prenatally [8–15]. With the growing knowledge about BWS and the currently applied molecular tests techniques in BWS, the number of positively tested BWS patients has increased including prenatal patients [8]. However, challenges exist for prenatal diagnosis of BWS. Mosaicism is a major challenge. In case of low-rate mosaicism, the negative result will be generated and the suspected cases will escape the detection. Since there is variable degree of methylation in different tissues of imprinting disease patients, to test another tissue (fibroblasts, buccal cells) in prenatal diagnosis of BWS is not easy to achieve. Another challenge is that the diagnostic work-up in BWS needs multi-method approaches and knowledge of limitations of the applied because of the clinical heterogeneity and the molecular complexity of the disorder [16].

In patient 2, aCGH test revealed a 2.5 Mb duplication at 11p15.5–15.4 and a 228.8Kb microdeletion at 11p15.5. This terminal deletion includes the *SIRT3, PSMD13, NLRP6, ATHL1, IF1TM1, IF1TM3, B4GALNT4, PKP3, SIGIRR,* and *ANO9* genes. The NAD-dependent deacetylase sirtuin-3 (*SIRT3*) gene, a member of the sirtuin family of protein, is an important regulator of cell metabolism. *SIRT3* plays a key role in cellular respiration, metabolism, aging-related disease and cancer [17]. The *NLRP6* gene, a member of the nucleotide-binding oligomerization domain-like receptor (*NLR*) family member, has been implicated in inflammasome signaling to activate caspase-1. *NLRP6* plays critical role for protection against inflammation-related colon tumorigenesis [18]. Patient 2 had the de novo duplication at 11p15 and can be classified into low tumor risk [19]. Interestingly, the clinical feature of macroglossia resolves spontaneously. It has been observed that some features mitigate during the growth of BWS patient as reported in previous studies [3, 11, 20]. For patient 2, physical examination should be performed routinely for potential intellectual disability and the possible clinical effect involving the deleted genes.

**Fig. 3:**
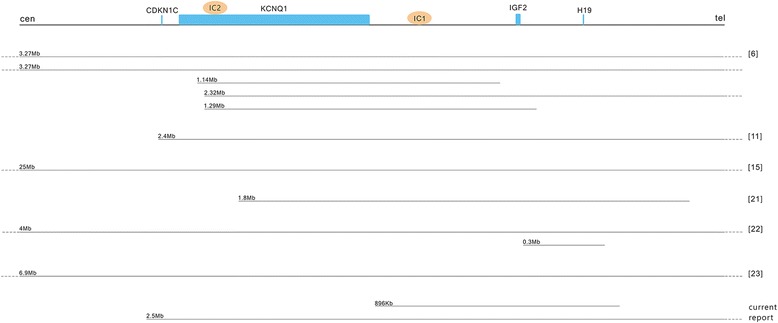
Six reports with known size of paternally derived duplications involving in BWS. Duplication segments were depicted in solid line and the dash line represents the exceeding length. The citation number is noted on the right. Drawing is not to scale

Review of literature found six reports with known size of paternally derived duplications involving in BWS [6, 9, 14, 21–23]. The duplication segments ranged in size from 0.3 Mb to 25 Mb (Fig. 3). The breakpoints position of duplication segment less than 2.0 Mb differ while invariably fall within the IC1 or IC2 region, which suggests that the genomic instability in this region may directly related to the genomic structure. The non-recurrent genomic replication is probably mediated by the non-allelic homologous recombination (NAHR) which results in the overexpression of *IGF2*. The previous studies have revealed that recurrent CNVs are characterized by higher GC content and hot spot motif [24]. The methylation aberration of *KCNQ1OT1:TSS DMR* or *H19/IGF2:IG DMR*, and the breakpoints sequence analysis should be further studied in this highly genomic instability region. The identification of more cases with duplication at 11p15.5 imprinted domain may bring new insights into the regulation mechanism of genomic imprinting at both IC1 and IC2 [9]. Furthermore, chromosome duplications or deletions have been infrequently reported in North American and European patients. It is estimated that about 1–2% of the BWS patients have a chromosome duplication or a rearrangement (inversion or translocation) [5]. A recent study of 47 Japanese BWS patients revealed a significantly higher frequency of chromosome abnormality (13%) and extremely low frequency of *H19/IGF2:IG DMR* hypermethylation (0%). The data suggested that susceptibility to epigenetic and genetic alterations leading to BWS varied according to ethnicity [25]. A recent study in a serial of over 400 BWS cases also indicated that copy number changes in the 11p15.5 region contributed significantly to the etiology of the BWS [6]. Large case series to systematically evaluate the subtype frequency of BWS in Chinese population is essential to find out the underlying environmental or genetic factors. The existence of low copy repeats and segmental duplications as hot-spot for copy number changes at 11p15.5 should be investigated further, especially in East Asian population [21, 26].

## Conclusion

In conclusion, we first reported two Chinese BWS patients caused by paternally derived de novo duplication at 11p15. More cases should be collected to study whether there is ethnicity genetic background difference for various subtypes of epigenetic-genetic alterations. Identifying the underlying genetic mechanisms causing BWS is important for informative genetic counseling, effective disease treatment, recurrence and tumor risk management.
